# *In vitro* exposure to polystyrene microplastics exerts oocyte toxicity through cumulus cells damage in the sheep model

**DOI:** 10.3389/fvets.2026.1771581

**Published:** 2026-03-02

**Authors:** Letizia Temerario, Andrea Podda, Luisa Bogliolo, Antonella Mastrorocco, Maria Carmela Ferrante, Pierfrancesco Pinto, Maria Elena Dell’Aquila, Nicola Antonio Martino

**Affiliations:** 1 Department of Biosciences, Biotechnology and Environment, University of Bari Aldo Moro, Bari, Italy; 2 Department of Veterinary Medicine, University of Sassari, Sassari, Italy; 3 Department of Veterinary Medicine and Animal Productions, University of Naples Federico II, Naples, Italy; 4 Prevention Department of ASL BA, Animal Origin Food Hygiene Service, Bari, Italy

**Keywords:** microplastics, oocyte, *in vitro* maturation, embryo, gene expression, apoptosis, oxidative stress, cytoskeleton

## Abstract

**Introduction:**

In recent years, the widespread environmental presence of microplastics (MPs) has raised major concerns regarding animal and human health, including potential risks to reproductive function and offspring.

**Methods:**

This study aimed to evaluate the effects of increasing concentrations of polystyrene MPs (PS-MPs; 0, 5, 50, or 100 μg/mL) on ovine cumulus–oocyte complexes (COCs) during *in vitro* maturation (IVM). Fluorescent microspheres were used for uptake assessment into COCs and cumulus cells (CCs) monolayers, whereas non-fluorescent PS-MPs were employed to evaluate potential toxic effects induced on CCs and oocytes.

**Results:**

As regards CCs, increased PS-MPs uptake was highlighted at the highest exposure concentration (100 μg/mL), whereas no significant differences were observed in oocyte intracellular fluorescence intensity, compared to the control. The bioaccumulation increment in CCs monolayers was already visible after 6 h, both at 5 and 100 μg/mL, and confirmed at 24 h. The real-time PCR analysis in CCs revealed significant reductions in the expression levels of genes involved in antioxidant defense and alterations in those implicated in apoptosis. Finally, the TUNEL assay revealed a dose-dependent increase in CCs apoptotic index. Consequently, PS-MPs exposure impaired oocyte meiosis resumption by significantly reducing the maturation rates, particularly at 50 and 100 μg/mL, whereas no effects were observed at 5 μg/mL. Oocyte intracellular reactive oxygen species levels were significantly increased at all concentrations, whereas no differences in mitochondrial membrane potential were detected. The percentages of oocytes with abnormal configurations of meiotic spindle and cortical F-actin were found to be significantly increased, regardless of concentration. Finally, the cleavage rate was significantly reduced in oocytes exposed to 50 μg/mL, whereas no differences were found in the blastocyst rate at both 50 and 5 μg/mL.

**Discussion:**

In conclusion, *in vitro* exposure of sheep COCs to PS-MPs during IVM reduced oocyte quality and developmental potential through alterations induced in the CCs, which turned out to be the main target of these environmental contaminants.

## 1 Introduction

Currently, there is growing recognition of the role that environmental pollutants exert in declining human and animal fertility (1–8). Among them, plastic waste represents an escalating global health issue due to the increasing accumulation of small plastic particles, known as micro- and nano-plastics (MPs and NPs), in all environmental compartments, including water, soil, atmosphere, and food chains (1, 9–14). Plastic pollution primarily originates from industrial and agricultural anthropic activities, inadequate waste management, environmental calamities, and finally the common use of littering of single-use plastics, packaging, and food waste (1, 9–14). Once released into the environment, plastic undergoes degradation through physical, chemical, and biological processes (15), thus leading to the breakdown of larger plastic debris into MPs, ranging from 100 nm to 5 mm in diameter, and NPs between 1 and 100 nm (1, 10–14, 16, 17). Physically, these particles vary in size, color, and shape, ranging from fragments, foams, and beads to fibers, films, and flakes (1, 10–14). Chemically, their composition is variable and can include materials such as polystyrene (PS), polyethylene (PE), polypropylene (PP), polyvinyl chloride (PVC), and polyethylene terephthalate (PET) (1, 10–14). Moreover, the presence of toxic metals or plastic additives, adhering to their surfaces, can additionally influence the properties and effects of these particles (1, 11).

Animals and humans can be exposed to MPs and NPs through multiple pathways, including ingestion, inhalation, and dermal contact (1, 10–14). Exposure to these particles poses several potential health risks, including those on reproductive function and offspring, exerted through multiple mechanisms, such as oxidative stress, inflammation, and apoptosis (18). MPs and NPs are able to pass through the blood–follicle barrier and to potentially penetrate the oocyte through gap junctions or cross the zona pellucida, resulting in impairment of oocyte maturation, fertilization, and embryo development (1, 10, 13, 14, 18). Moreover, these particles are known to induce apoptosis of granulosa cells, reduce ovarian follicle reserve, and accumulate in the ovary and uterus with subsequent fibrosis (1, 10, 13, 14). It has also been reported that MPs can intensify the toxic effect of environmental pollutants or endocrine disruptors on reproduction (19). In addition, potential carry-over effects on the next generation are reported due to the ability of MPs and NPs to pass through the blood–placenta barrier (1, 12–14).

Current knowledge on the potential effects of MPs and NPs mainly derives from studies on aquatic and terrestrial species of vertebrates and invertebrates (1, 20–22) and, more recently, from rodents (1). On the contrary, research in farm animals is limited up to now, although their exposure is documented and they represent interesting translational models for human reproduction (1, 13, 14). In particular, among them, the sheep has become a valuable experimental model due to the similarities with human ovarian structure, oocyte size, and follicle development dynamics. Specifically, oocytes retrieved from juvenile animals, representing a widely available source of biological material due to lamb meat consumption, are interesting for research in the pediatric age (23).

This study aimed to evaluate the effects of PS-MPs on ovine cumulus–oocyte complexes (COCs), exposed during *in vitro* maturation (IVM), by assessing uptake into COC and cumulus cell (CC) monolayers, CC’s quantitative gene expression and apoptotic index, and oocyte maturation and developmental competence.

## 2 Materials and methods

**Figure 1:**
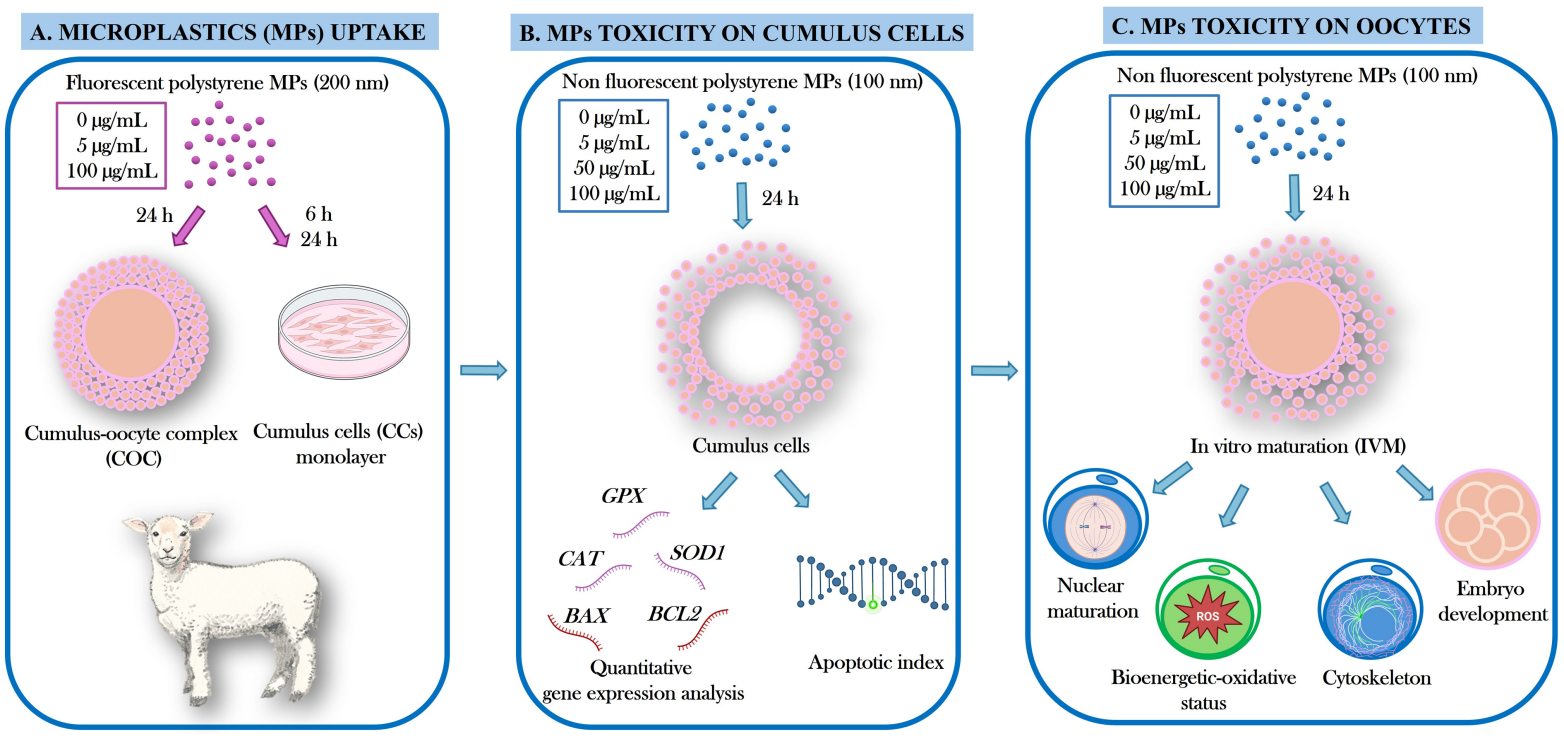
Experimental design flowchart. In the present study, the effects of exposure to polystyrene microplastics (PS-MPs) during *in vitro* maturation (IVM) on sheep cumulus–oocyte complexes (COCs) were evaluated through uptake assessment into COCs and cumulus cells (CCs) monolayers **(A)**, CCs quantitative gene expression and apoptotic index **(B)**, and oocyte maturation and developmental competence **(C)**.

The experimental design flowchart is presented in Figure 1.

### 2.1 Chemicals

All chemicals were purchased from Sigma-Aldrich (Milan, Italy), unless otherwise indicated.

### 2.2 Collection of ovaries and retrieval of cumulus–oocyte complexes (COCs)

Ovaries were collected at a local slaughterhouse (Surace Carne s.r.l., Noci, Bari) from prepubertal lambs (<3 months of age) subjected to routine veterinary inspection and transported to the laboratory at room temperature within 2–4 h from slaughter. The lamb’s prepubertal status was later confirmed at the ovarian level, in the laboratory, by evaluating the absence of growing follicles and corpora lutea. For the retrieval of COCs, the slicing technique was employed. The follicular contents were released into 60-mm sterile Petri dishes containing phosphate-buffered saline (PBS). COCs were selected using a Nikon SMZ 1500 optical stereomicroscope (Nikon Instruments, Firenze, Italy). Only those exhibiting at least three intact layers of CCs and homogeneous cytoplasm were selected for *in vitro* culture (24).

### 2.3 COC exposure to polystyrene microplastics (PS-MPs) and *in vitro* maturation (IVM)

To evaluate the effects of PS-MPs on COC uptake and oocyte IVM, specific culture medium solutions were prepared by incorporating commercially available PS beads (Bangs Laboratories Inc., Fishers, IN, USA) at several concentrations. Fluorescent microspheres conjugated with Flash Red (200 nm diameter) were used for uptake assessment, while those not conjugated to the fluorophore (100 nm diameter) were employed to assess oocyte and CCs toxicity. IVM medium was prepared, as previously described (24), based on TCM-199 medium with Earle’s salts, buffered with 5.87 mmol/L HEPES and 33.09 mmol/L sodium bicarbonate and supplemented with 0.1 g/L L-glutamine, 2.27 mmol/L sodium pyruvate, calcium lactate pentahydrate (1.62 mmol/L Ca^2+^, 3.9 mmol/L lactate), 50 μg/mL gentamicin, 20% (v/v) fetal calf serum (FCS), 10 μg/mL of porcine follicle-stimulating hormone and luteinizing hormone (FSH/LH; Pluset^®^, Calier, Barcelona, Spain) and 1 μg/mL 17-*β*-estradiol. On the day of experiments, PS-MPs stock solutions were diluted with IVM medium to cover a concentration range from 5 to 100 μg/mL, according to the experimental design. IVM medium without PS-MPs was used as a control. For each set of experiments, groups of 20–25 selected COCs were placed in four-well dishes (Nunc Intermed, Roskilde, Denmark) containing 400 μL of IVM, supplemented or not with PS-MPs, covered with an equal volume of pre-equilibrated lightweight mineral oil per well. IVM culture was performed for 22–24 h at 38.5 °C with 5% CO_2_ in a humidified incubator (24).

### 2.4 Exposure of CCs cultured in a single layer to PS-MPs

Considering the functions of CCs in COCs, the effects of exposure to PS-MPs were verified over time. Specifically, the bioaccumulation of fluorescent PS-MPs in the CCs after 6 and 24 h of incubation was evaluated in the same experimental conditions previously described for COCs. Recovered CCs were centrifuged at 300 *g* for 5 min, the supernatant was aspirated, and the pellet was resuspended in 100 μL of PBS. Meanwhile, 10 slides were prepared, coverslipped inside 2 plates with 35-mm diameter wells. Subsequently, CCs were adhered onto the slides, and Dulbecco’s Modified Eagle Medium (DMEM) culture medium, implemented with 20% (v/v) FCS, with or without PS-MPs, was added.

### 2.5 Assessment of fluorescent PS-MPs uptake

After IVM, COCs were washed in PBS and fixed in 4% paraformaldehyde (PFA) solution in PBS. Subsequently, COCs were washed a second time in PBS, mounted on slides in a drop of glycerol–PBS solution with a ratio of 3:1 (v/v), covered with cover slips, and sealed with nail polish. Finally, the slides were stored at 4 °C in the dark. To evaluate the uptake of fluorescent PS-MPs, COCs and CCs monolayers were observed under a Nikon C1/TE2000-U confocal laser scanning microscope (Nikon Instruments, Firenze, Italy) at 600x magnification under oil immersion. To detect Flash Red fluorescence, a 633-nm helium/neon laser beam and an LP-650 filter were used. The fluorescence intensities were measured on the equatorial plane, with the aid of the EZ-CI GoldVersion 3.70 image analysis software platform for the Nikon CI confocal microscope (Nikon Instruments, Firenze, Italy). A circular area was traced to delineate the cytoplasmic area of the oocytes, whereas a polygonal area was traced by dividing CCs into sectors. Instead, for the fluorescence intensity of the CCs plated in a monolayer, a polygonal area was traced for each CC of the group present in the framed field. The fluorescence intensity within the programmed scanning area (512 × 512 pixels) was recorded and expressed as arbitrary densitometric units (ADU). The evaluations of all samples were carried out under fixed scanning conditions with regard to laser energy, signal detection (gain), and pinhole size.

### 2.6 RNA extraction from CCs and quantitative real-time PCR

After IVM, COCs underwent a denuding procedure through the enzymatic action of hyaluronidase and mechanical pipetting of a Gilson pipette. Collected CCs were centrifuged at 400 *g* for 5 min at room temperature, and the resulting pellet was immediately stored at −80 °C. Total RNA extraction was performed using the Rneasy^®^ Plus Micro Kit (Qiagen, Hilden, Germany), following the manufacturer’s instructions (25). Single-stranded complementary DNA (cDNA) was synthesized by using the High-Capacity cDNA Reverse Transcription Kit (Applied Biosystems, Foster City, CA, USA) in a thermal cycler (Eppendorf, Hamburg, Germany) according to the following thermal program: (1) primers annealing at 25 °C for 10 min, (2) DNA polymerization at 37 °C for 120 min, and (3) enzyme deactivation at 85 °C for 5 min. Real-time PCR was used to perform gene expression analysis. For each sample, the reaction mix (20 μL) contained: 11 μL SYBR Green PCR Master Mix powerup (Applied Biosystems, 2x), 1 μL forward primer (Table 1), 1 μL reverse primer (Table 1), 1 μL cDNA, and RNase-free water up to the final volume. Each reaction was performed using a StepOne thermal cycler (Applied Biosystems, Foster City, CA, USA) with the following thermal cycling parameters: (1) 40 cycles of denaturation at 95 °C for 15 s each, (2) annealing at 60 °C for 1 min, (3) extension at 60 °C for 1 min. The specificity of each primer was confirmed through melting curve analysis. Each amplification was performed in duplicate, and the relative quantification of gene expression was conducted using the 2^-ΔΔCt^ method (Livak method) using *β*-actin as the housekeeping gene.

**Table 1:** Primer sequences validated by real-time PCR.

Symbol	Gene name	Identification number	Primer sequence (5′ → 3′)	Annealing temperature (°C)
ACTB	Actin Beta	NM_001009784	CCCTGGAGAAGAGCTACGAG TAGTTTCGTGAATGCCGCAG	59
SOD1	Superoxide dismutase 1	NM_001145185	GGCAATGTGAAGGCTGACAA TGCCCAAGTCATCTGGTCTT	59
CAT	Catalase	GQ421282	ACGCCTGTGTGAGAACATTG AGCCATACTCAGGATGGACA	59
GPX	Glutathione peroxidase	GAAI01007125	ACCCAGATGAATGACCTGCA TCGGACGTACTTCAGGCAAT	59
BCL2	B cell lymphoma 2	DQ15929	ATGACCGAGTACCTGAACCG GGAGAAATCAAACAGGGGCC	59
BAX	BCL2-associated X protein	XM_004015363	AAGAAGCTGAGCGAGTGTCT AAAACATTTCAGCCGCCACT	59

### 2.7 Terminal deoxynucleotidyl transferase-mediated dUTP Nick-end labeling (TUNEL)

For the TUNEL assay, CCs were centrifuged at 300 *g* for 5 min, and the resulting pellet was used to assess DNA fragmentation (26) using the Click-iT^®^ Plus TUNEL Assay (Invitrogen, Carlsbad, CA, USA) following the manufacturer’s instructions. Briefly, CCs were fixed in 4% PFA for 15 min at room temperature and then permeabilized with 0.5% Triton X-100 for 20 min. After washing with deionized water, CCs were incubated in 50 μL drops of the TUNEL reagent in the dark for 1 h at 37 °C in a humidified atmosphere. The total nuclei were stained with 2.5 μg/mL Hoechst 33258 in a 3:1 (v/v) glycerol/PBS solution, mounted onto microscope slides, covered with a coverslip, sealed with nail polish, and stored at 4 °C in the dark. CCs were observed under a Nikon E-600 fluorescence microscope equipped with excitation filters of 365 nm for Hoechst 33258 and 495 nm for TUNEL staining. For each condition, approximately 15 different fields were examined to evaluate at least 100 cells in total. The apoptotic index was determined as the percentage of TUNEL-positive cells relative to the total number of cells stained with Hoechst 33258. Fluorescence quantification was performed by manually selecting the area of each TUNEL-positive cell (27).

### 2.8 Oocyte mitochondria and reactive oxygen species (ROS) staining after IVM

After IVM culture and COC denuding, oocytes were washed in PBS with 3% BSA and incubated for 30 min in the same medium containing 280 nmoL/L of MitoTracker Orange CMTM Ros (Thermo Fisher Scientific, Waltham, MA, USA) at 38.5 °C under 5% CO_2_ in air (28). Then, the cells were washed in PBS with 0.3% BSA and incubated in the same medium with 10 μM 2′,7′-dichlorodihydrofluorescein diacetate (H_2_DCF-DA) for 15 min at 38.5 °C under 5% CO_2_ in air for intracellular reactive oxygen species (ROS) detection (28, 29). After incubation, oocytes were washed in pre-warmed PBS without BSA and fixed overnight in 4% PFA at 4 °C.

### 2.9 Oocyte nuclear chromatin evaluation after IVM

To evaluate nuclear chromatin status, fixed oocytes in PFA were stained with 2.5 μg/mL Hoechst 33258 in a 3:1 (v/v) glycerol/PBS mixture, mounted on microscope slides, and stored at 4 °C in the dark. Slides were analyzed using an epifluorescence microscope (Nikon Eclipse 600; ×400 magnification) equipped with a B-2A filter (excitation 346 nm/emission 460 nm). Oocytes were classified according to their meiotic stage as germinal vesicles (GV), metaphase to telophase I (MI to TI), or metaphase II (MII) with the first polar body (PB) extruded. Oocytes displaying one pronucleus with extruded PB, irregular chromatin clumps, absence of chromatin, or a multipolar meiotic spindle were categorized as abnormal (28).

### 2.10 Mitochondrial distribution pattern and intracellular ROS localization assessment

To detect and localize mitochondria and ROS, mature oocytes were observed using a Nikon C1/TE2000-U laser scanning confocal microscope (Nikon Instruments, Firenze, Italy) equipped with the Apo 60×/NA 1.40 Nikon Plan objective in oil immersion. A 543 nm helium/neon laser and a G-2A filter were used to detect the MitoTracker Orange CMTM Ros (551 nm excitation and 576 nm emission). A 488-nm argon ion laser and a B-2A filter were used to detect dichlorofluorescein (DCF) (495 nm excitation and 519 nm emission). Oocytes were observed in 25 optical sections with a step size of 0.45 μm, thus allowing 3D distribution analysis. The mitochondrial distribution pattern was evaluated as “perinuclear and subcortical (P/S)”, index of cytoplasmic maturity, “finely granular”, typical of immature oocytes and “abnormal”, with irregular mitochondria distribution (24). Concerning intracellular ROS localization, oocytes with intracellular ROS diffused throughout the cytoplasm, together with areas/sites of mitochondria/ROS overlapping, were considered viable.

### 2.11 Quantification of bioenergetic/oxidative parameters

In each individual oocyte, MitoTracker and DCF fluorescence intensities and the Manders’ overlap coefficient (30), indicating the extent of mitochondria/ROS colocalization, were measured at the equatorial plane using the EZ-C1 Gold Version 3.70 image analysis software platform for a Nikon C1 confocal microscope. A circular area was drawn around the ooplasm for the quantification analysis. The fluorescence intensity within the scanned area was recorded, and 16-bit images were obtained. Mitochondrial membrane potential and intracellular ROS concentrations were recorded as the fluorescence intensity emitted by each probe and expressed as arbitrary densitometric units (ADUs). Variables related to fluorescence intensity, such as laser energy, signal detection (gain), and pinhole size, were maintained at constant values for all measurements. In the mitochondria/ROS colocalization analysis, threshold levels were kept constant at 10% of the maximum pixel intensity for all measurements.

### 2.12 Immunofluorescence detection of cytoskeletal

Metaphase II (MII) oocytes were fixed for 1 h at 37 °C in a microtubule-stabilizing buffer and subsequently stored at 4 °C in blocking solution (31). Samples were incubated overnight at 4 °C with a mixture of mouse monoclonal antibodies directed against *α*-tubulin (1:1000 dilution) and *β*-tubulin (1:100 dilution). Immunolabeling was then performed using donkey anti-mouse secondary antibodies conjugated with fluorescein isothiocyanate (FITC-Alexa Fluor 488; 1:100 dilution; Life Technologies, Invitrogen, Carlsbad, CA, USA), in combination with rhodamine–phalloidin (1:150 dilution; Invitrogen, Carlsbad, CA, USA), for 1 h at room temperature. Nuclear chromatin was counterstained with Hoechst 33258 (10 μg/mL). Confocal imaging of meiotic spindle microtubules, chromatin organization, and cortical F-actin architecture was carried out using a laser scanning confocal microscope (Leica TCS SP5, Leica, Wetzlar, Germany) equipped with Ar/He/Ne lasers and a 40 × oil-immersion objective. Hoechst 33258, FITC, and rhodamine–phalloidin were excited at 358 nm, 488 nm, and 551 nm, respectively, with fluorescence emission signals collected at 461 nm, 550 nm, and 595 nm. Oocytes were optically sectioned along the Z-axis, and cortical F-actin images were acquired at the equatorial plane. Oocytes were classified based on meiotic spindle morphology (normal symmetrical barrel-shaped spindles versus abnormal disorganized, clumped, or dispersed structures), chromosomal alignment (proper alignment versus misalignment or dispersion at the metaphase plate), and cortical F-actin distribution (continuous, uniformly distributed subplasmalemmal F-actin layer versus irregular or discontinuous organization) (31). The proportion of oocytes displaying normal or aberrant spindle, chromatin, and cortical F-actin configurations was quantified for each experimental group.

### 2.13 Oocyte parthenogenetic activation (PA) and *in vitro* embryo culture (IVEC)

To evaluate the effects of PS-MPs exposure on developmental competence, oocytes were parthenogenetically activated (PA) after IVM with 5 μM ionomycin in TCM-199 for 5 min, followed by 4 h of culture in TCM-199 with 2 mM 6-dimethylaminopurine (6-DMAP) in a humidified atmosphere with 5% CO_2_ at 38.5 °C (32). PA oocytes underwent *in vitro* embryo culture (IVEC) for 7 days in four-well dishes containing 500 μL/well of synthetic oviductal fluid medium (SOFM) with essential and non-essential amino acids at oviductal concentrations and 0.4% bovine serum albumin (BSA) under mineral oil in humidified atmosphere with 5% CO_2_, 5% O_2_, and 90% N_2_ at 38.5 °C (3). Embryo development was followed by conventional morphology assessment under phase contrast microscopy. The cleavage was evaluated morphologically after 24 and 48 h of culture, while development to the blastocyst stage was assessed on day 7 (32). Blastocysts were classified according to the expansion and hatching status as early (initiating blastocoelic cavity formation), cavitated (full blastocoelic cavity formation), expanded (increased size with blastocelic cavity greater than half of the embryo volume and thinner zona pellucida), hatching (beginning of the exit from the zona pellucida), or hatched (full exit from the zona pellucida). Moreover, blastocyst diameter was evaluated by Oosight™ Research Instruments as the distance between the outside borders of the trophectoderm. Finally, blastocyst formation was confirmed on the last day of the culture by observing blastomere nuclear chromatin under epifluorescence microscopy after staining with Hoechst 33258, as indicated before.

### 2.14 Statistical analysis

Oocyte chromatin configurations, mitochondria distribution patterns, cytoskeletal configurations, cleavage rates, blastocyst rates, and apoptotic index were compared between conditions using the Chi-square test. PS-MPs uptake, bioenergetic oxidative status parameters, and gene expression were compared using one-way analysis of variance (ANOVA) followed by Tukey’s multiple comparison test. Data were analyzed with GraphPad Prism (Software version 8.0.2, San Diego, CA, USA). Differences with *p <* 0.05 were considered statistically significant.

## 3 Results

### 3.1 Assessment of fluorescent PS-MPs uptake into sheep COCs and CCs monolayers

**Figure 2:**
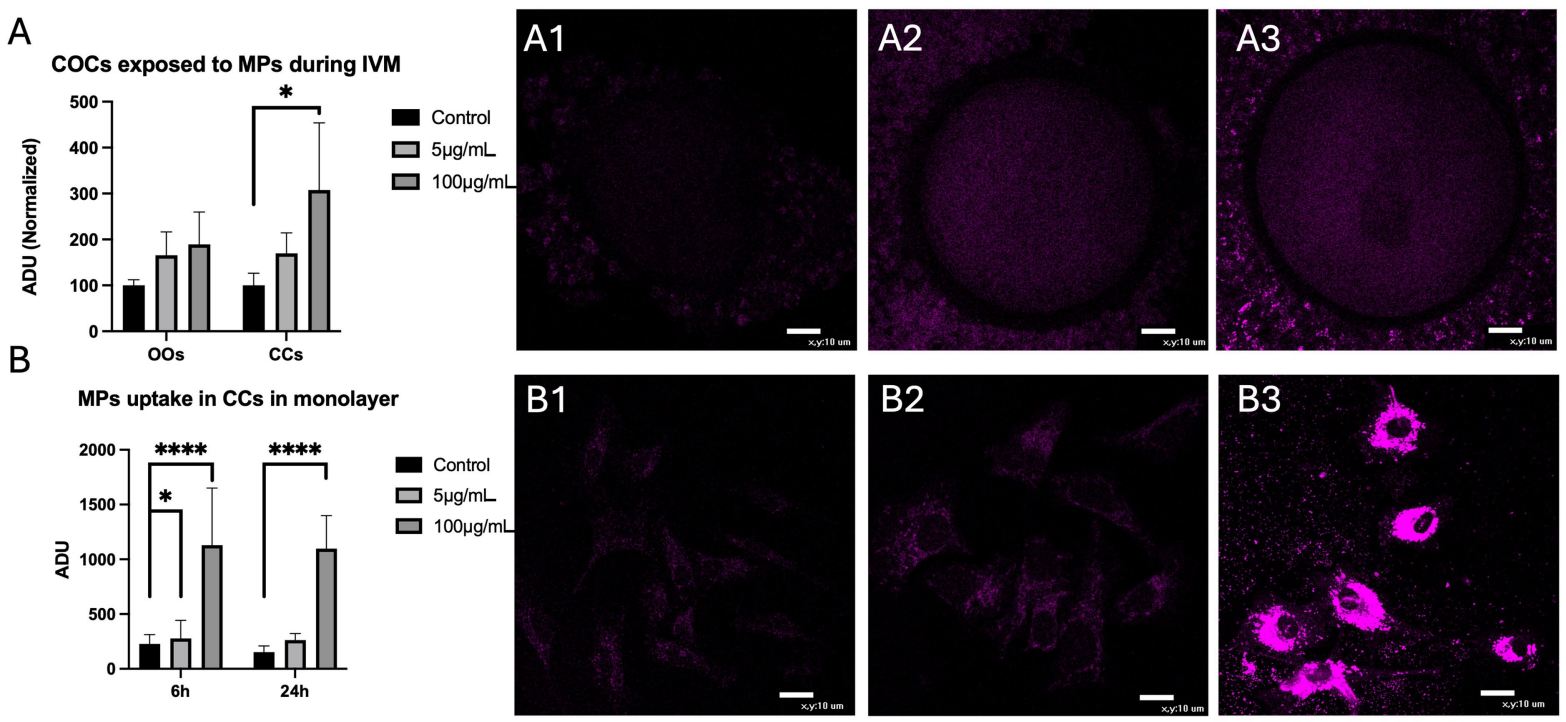
Assessment of PS-MPs uptake into oocytes (OOs) and cumulus cells (CCs) of sheep COCs after 24 h IVM **(A)** and CCs grown in monolayers after 6 and 24 h **(B)** of exposure to fluorescent 200 nm microparticles concentrated 0 μg/mL **(A1,B1)**, 5 μg/mL **(A2,B2)**, and 100 μg/mL **(A3,B3)**. One-way analysis of variance (ANOVA) followed by Tukey’s multiple comparison test: *p < 0.05; ****p < 0.0001. Scale bar = 10 μm.

The uptake of fluorescent 200 nm PS-MPs was assessed on a total of 30 ovine COCs at the end of IVM culture. Only the lowest and highest PS-MPs concentrations were used for this test (5 μg/mL and 100 μg/mL, respectively). For oocytes, no significant differences in intracellular fluorescence intensity were observed in comparison to the control condition, regardless of PS-MPs concentration. In contrast, for CCs, increased PS-MPs uptake was observed after exposure to the highest tested concentration (*p <* 0.05; Figure 2A). Bioaccumulation of fluorescent PS-MPs over time in CCs was analyzed in monolayer-cultured CCs. Increased PS-MPs bioaccumulation was observed already after 6 h of *in vitro* exposure, both at 5 μg/mL (*p <* 0.05) and 100 μg/mL (*p <* 0.0001) concentrations compared to the control condition. After 24 h of exposure, the intracellular uptake of PS-MP was confirmed at the highest concentration (*p <* 0.0001; Figure 2B).

### 3.2 Effects of PS-MPs exposure on cumulus cell gene expression

**Figure 3:**
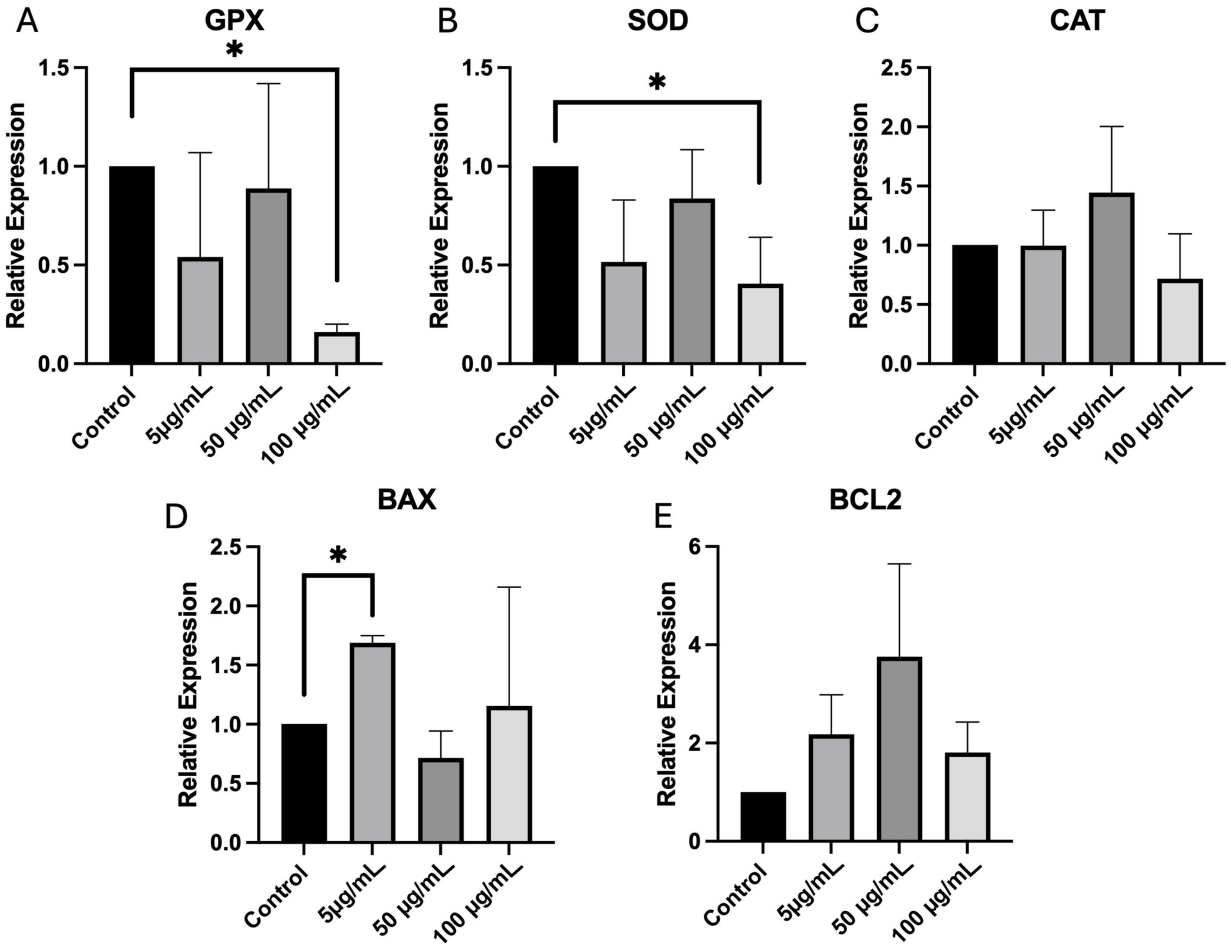
Effects of PS-MPs on oxidative stress and apoptosis in CCs. Relative expression of GPX **(A)**, SOD **(B)**, CAT **(C)**, BAX **(D)**, and BCL2 **(E)** genes, validated using real-time PCR, in CCs isolated from COCs exposed during IVM. One-way analysis of variance (ANOVA) followed by Tukey’s multiple comparison test: *p < 0.05.

The real-time PCR analysis of CCs isolated from 356 COCs exposed to PS-MPs during IVM revealed significant alterations in the expression of genes related to oxidative stress and apoptosis across the different treatment groups (Figure 3). The expression levels of GPX and SOD, key enzymes involved in antioxidant defense, were reduced at the concentration of 100 μg/mL (*p <* 0.05). CAT expression did not vary at any tested concentration. Regarding apoptosis-related gene expression, BAX, a proapoptotic gene, was significantly upregulated only at 5 μg/mL (*p <* 0.05), whereas BCL2 expression did not reach statistical significance.

### 3.3 Effects of PS-MPs exposure on cumulus cell apoptotic index

**Figure 4:**
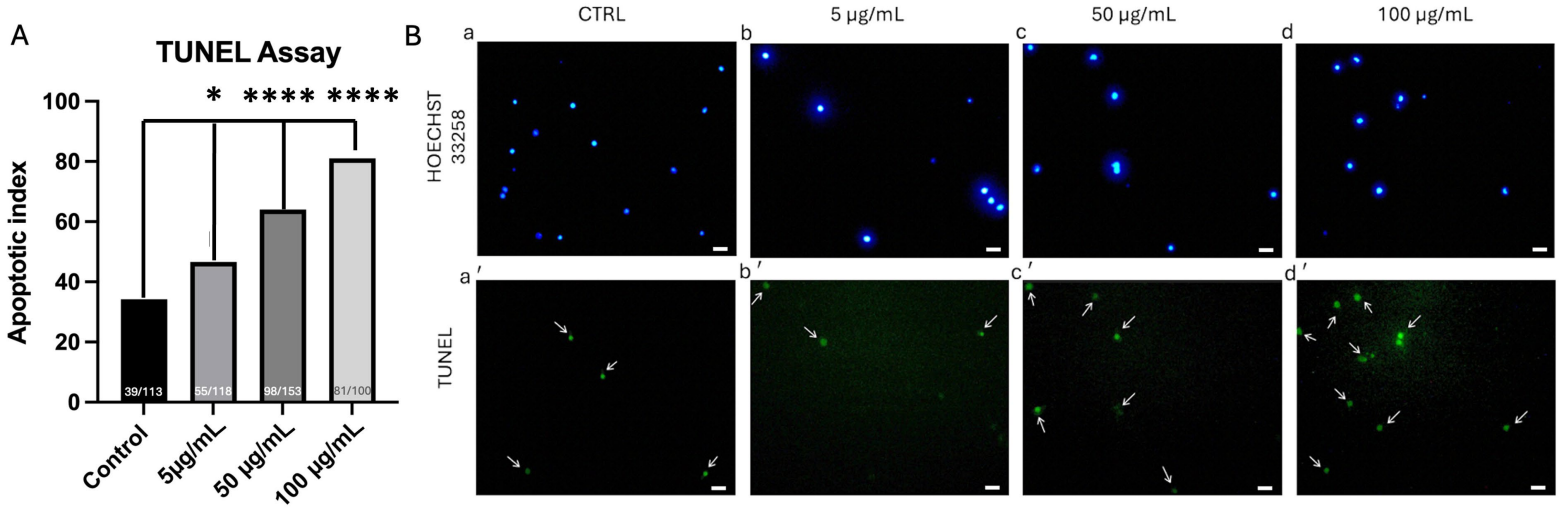
Effects of PS-MPs on CCs apoptosis by terminal deoxynucleotidyltransferase-mediated dUTP nick-end labeling (TUNEL) assay. Bar graphs showing the percentages of TUNEL-positive (with fragmented DNA) CCs after COC exposure to different PS-MPs concentrations **(A)**. The number of analyzed CCs per experimental condition is indicated at the bottom of each graph. Chi-squared test: within each column, different superscripts indicate statistically significant differences: *p < 0.05; ****p < 0.0001. Representative images of CCs observed after IVM in the presence of PS-MPs **(B)** at 0 g/mL **(a,a’)**, 5 g/mL **(b,b’)**, 50 g/mL **(c,c’)**, and 100 g/mL **(d,d’)**. Scale bar indicates 10 m.

The TUNEL assay revealed a dose-dependent increase in positive CCs (green-stained cells with fragmented DNA) isolated from a total of 68 COCs exposed to PS-MPs during IVM (46.6% at 5 μg/mL, *p <* 0.05; 64% at 50 μg/mL, *p <* 0.0001; and 81% at 100 μg/mL, *p <* 0.0001) compared to the control condition, which showed only 34% of TUNEL-positive cells (Figure 4).

### 3.4 Effects of PS-MPs exposure on oocyte nuclear maturation

The evaluation of the effects of non-functionalized 100 nm PS-MPs was performed on a total of 426 cultured COCs after IVM (5 replicates). As shown in Table 2, exposure to PS-MPs impaired meiosis resumption, particularly at higher tested concentrations. The percentage of oocytes reaching the MII + PB stage was significantly lower at 50 and 100 μg/mL PS-MPs (*p <* 0.01), whereas exposure to 5 μg/mL did not affect the maturation rate compared to untreated oocytes. These data were associated with a significant increase in the GV rates, particularly at 50 μg/mL and 100 μg/mL (*p <* 0.05). Notably, no significant differences were observed in the rates of abnormal chromatin configurations at all tested exposure concentrations.

**Table 2:** Effects of PS-MPs exposure during IVM on oocyte nuclear maturation.

PS-MP concentration (μg/mL)	Cultured COCs N.	Evaluated oocytes N.	Nuclear chromatin configurations N. (%)			
			GV	MI to TI	MII + PB	Abnormal
0 (Control)	100	88	12 (13.6)^𝑎^	8 (9.1)^𝑎^	56 (63.6)^a^	8 (9.1)
5	94	78	15 (19.2)	5 (6.4)	46 (59)	5 (6.4)
50	111	99	28 (28.3)^𝑏^	14 (14.1)	44 (44.4)^c^	8 (8.1)
100	121	110	29 (26.4)^𝑏^	21 (19.1)^𝑏^	45 (41)^c^	12 (10.9)

PS-MPs, Polystyrene Microplastics; IVM, *In Vitro* Maturation; COC, Cumulus-Oocyte Complex; GV, Germinal Vesicle; MI, Metaphase I; TI, Telophase I; MII, Metaphase II; PB, Polar Body. Comparisons for meiotic stages, between each tested condition versus control: Chi-squared test: within each column, different superscripts indicate statistically significant differences: ^a,b^*p <* 0.05; ^a,c^*p <* 0.01.

### 3.5 Effects of PS-MPs exposure on oocyte cytoplasmic quality

Qualitative and quantitative parameters of bioenergetic/oxidative status were assessed in oocytes reaching nuclear maturation after IVM as a measure of cytoplasmic quality and developmental competence. Regarding the qualitative parameter, the rates of MII + PB oocytes displaying heterogeneous P/S distribution patterns (43% for control [9/21], 43% for 5 μg/mL [10/23], 24% for 50 μg/mL [4/17], and 31% for 100 μg/mL [5/16]) were not significantly affected by PS-MPs exposure.

**Figure 5:**
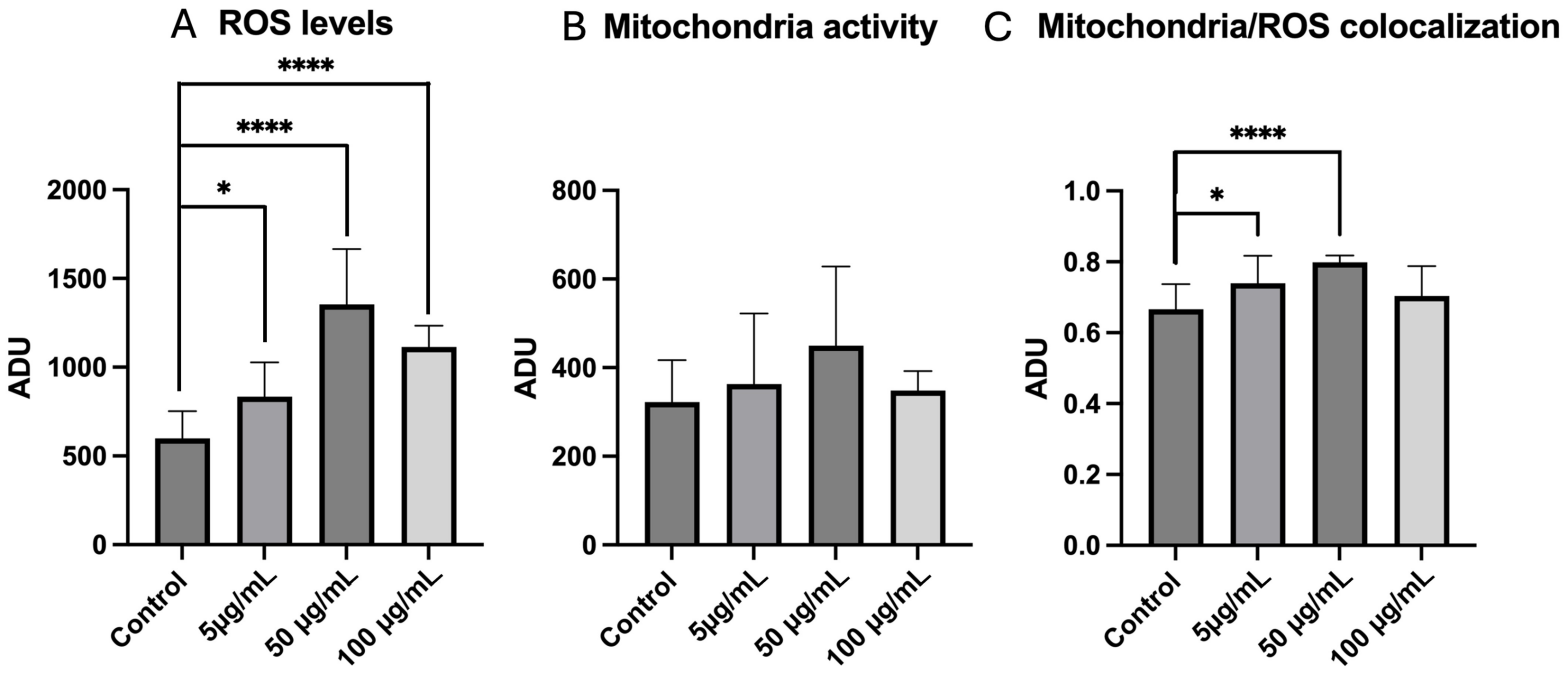
Effects of PS-MPs exposure during IVM on mitochondrial activity, intracellular ROS levels, and mitochondria/ROS colocalization in sheep mature oocytes. MitoTracker Orange CM™ Ros fluorescence intensity levels **(A)**, dichlorofluorescein (DCF) **(B)**, and mitochondria/ROS overlap coefficients **(C)** are expressed in arbitrary densitometric units (ADU). The number of oocytes analyzed by LSCM per experimental condition is indicated at the bottom of each histogram. One-way analysis of variance (ANOVA) followed by Tukey’s multiple comparison test: *p < 0.05; ****p < 0.0001.

The quantification of bioenergetic/oxidative status parameters revealed increased intracellular ROS levels at all PS-MPs exposure concentration compared to the control group (*p <* 0.05 for 5 μg/mL, *p <* 0.0001 for 50 μg/mL and 100 μg/mL; Figure 5A) whereas no differences were observed for mitochondrial membrane potential (Figure 5B). Finally, mitochondria/ROS colocalization showed a significant increase in oocytes exposed to 5 μg/mL (*p <* 0.05) and 50 μg/mL (*p <* 0.0001) (Figure 5C).

### 3.6 Effects of PS-MPs exposure on oocyte cytoskeletal organization

**Figure 6:**
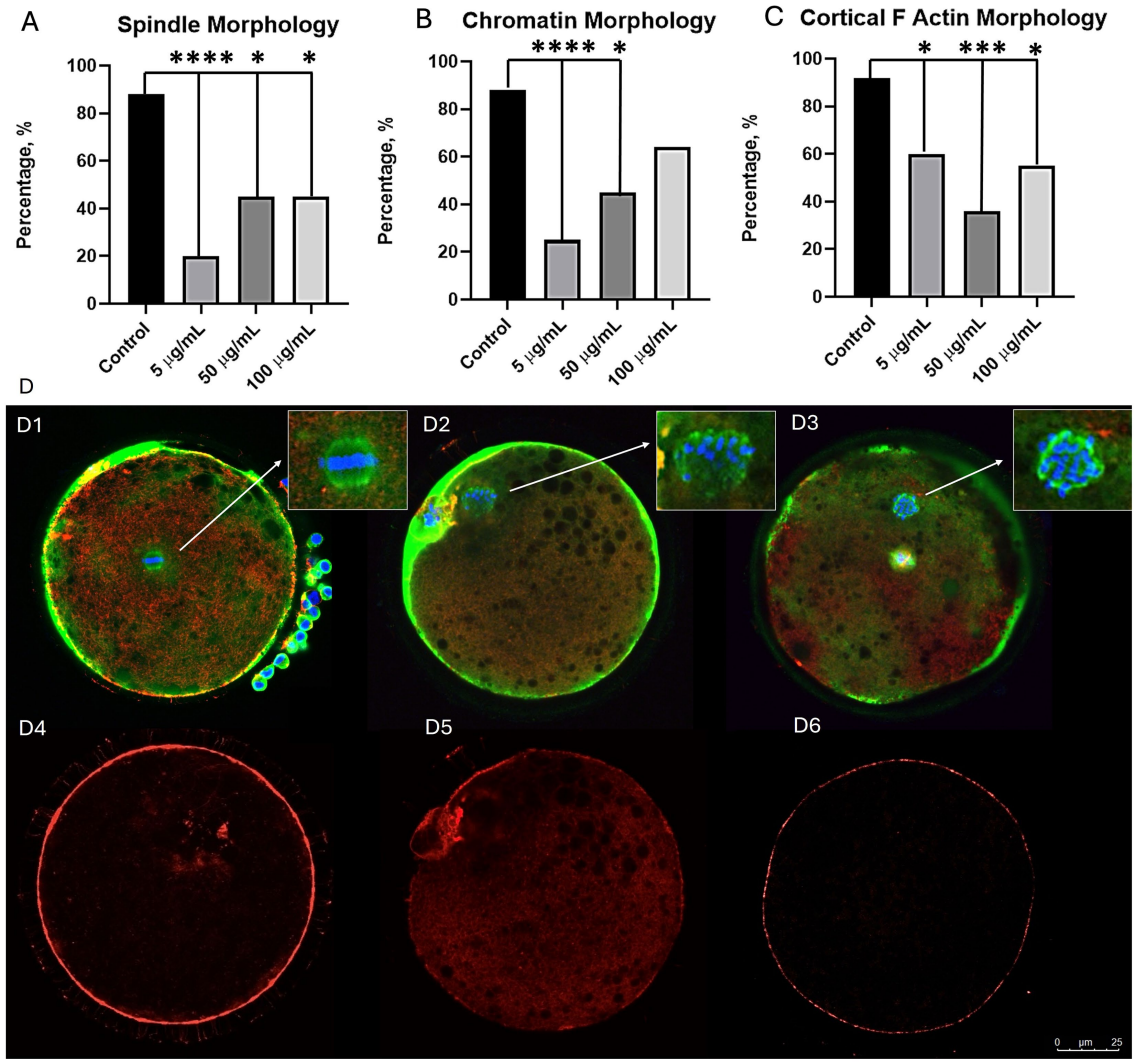
Effects of PS-MPs exposure on oocyte chromatin and cytoskeleton morphology. Graphs showing the percentage of oocytes with normal chromatin **(A)**, meiotic spindle **(B)**, and F-actin configuration **(C)**. Chi-squared test: *p < 0.05; ***p < 0.0001; ****p < 0.00001. Representative LSCM images representing normal chromatin (blue) and spindle (green) configuration **(D1)**, abnormal chromatin and spindle configuration **(D2,D3)**, normal cortical -actin (red) **(D4)**, and abnormal cortical -actin **(D5,D6)**. Scale bar = 25 m.

Oocyte exposure to PS-MPs resulted in perturbations in meiotic spindle assembly, chromosome alignment, and actin filament distribution. Indeed, the rate of oocytes exhibiting a classical barrel-shaped meiotic spindle structure was lower compared to untreated oocytes, regardless of PS-MPs concentration (*p <* 0.0001 for 5 μg/mL, *p <* 0.05 for 50 μg/mL, and *p <* 0.05 for 100 μg/mL) (Figure 6A). The rate of oocytes with correctly aligned chromosomes was also found to be reduced, particularly after exposure to 5 and 50 μg/mL (*p <* 0.0001 and *p <* 0.05, respectively) (Figure 6B). Finally, the percentage of oocytes with normally distributed actin filaments was reduced at all PS-MPs concentrations (*p <* 0.05 for 5 μg/mL, *p <* 0.001 for 50 μg/mL and *p <* 0.05 for 100 μg/mL), with oocytes displaying irregular staining in the area beneath the oolemma or spots staining within the ooplasm (Figure 6C).

### 3.7 Effects of PS-MPs exposure on oocyte developmental competence

The evaluation of the effects of PS-MPs exposure on developmental competence was performed on a total of 151 oocytes (three replicates). Only the lowest and intermediate PS-MPs concentrations were used for this test (5 μg/mL and 50 μg/mL, respectively). After 24 h of PA, the total cleavage rate was significantly reduced in oocytes exposed to 50 μg/mL (*p <* 0.01; Table 3), whereas no differences were found at 5 μg/mL compared to the control. After 48 h, no differences were observed in the cleavage rate, regardless of PS-MPs concentration (Table 3). After 7 days of IVEC, the blastocyst formation rate was not statistically different among conditions (Table 3). Regarding blastocyst quality, no differences were observed in either the mean diameter or the total number of nuclei, among experimental groups (Table 3). Nevertheless, hatched blastocysts were only obtained in the control group.

**Table 3:** Effects of PS-MPs exposure during IVM on oocyte developmental competence.

PS-MPs concentration (μg/mL)	PA oocytes N.	Cleavage rate at 24 h N. (%)	Cleavage rate at 48 h N. (%)	Blastocyst rate N. (%)	Blastocyst diameter Mean (μm) ± s.d.	Blastocyst nuclei Mean ± s.d.
0 (Control)	52	26 (50)^a^	30 (58)	7 (23)	193.3 ± 67.8	79 ± 29
5	49	22 (45)	31 (63)	3 (10)	176.4 ± 40.6	83 ± 31
50	50	11 (22)^c^	25 (50)	3 (12)	179.8 ± 34.7	102 ± 34

PS-MPs, Polystyrene Microplastics; IVM, In Vitro Maturation; PA, Parthenogenetically Activated. Comparisons for cleavage and blastocyst rates, between each tested condition versus control: Chi-squared test: within each column, different superscripts indicate statistically significant differences: ^a,c^*p <* 0.01. Comparisons for blastocyst diameter and nuclei, between each tested condition versus control: One-way analysis of variance ANOVA followed by Tukey’s multiple comparison test: not significant.

## 4 Discussion

In the present study, the effects of different concentrations of PS-MPs were evaluated on the cumulus–oocyte complex in the ovine model. PS-MPs were chosen based on their global prevalence (9, 18, 19, 33) and environmental persistence, which results from their slow degradation (9). Furthermore, this polymer has been reported as the second most prevalent type detected in human blood samples, further supporting this choice (34).

The first step was to evaluate PS-MPs uptake into COCs and CC monolayers. Previous studies have highlighted the ability of MPs to enter cells and accumulate in animal tissues, depending on particle size, concentration, and exposure time (9, 10, 13, 14). Regarding the female reproductive system, PS-MPs presence is well-documented in the ovary (9, 10, 13, 14, 35), whereas little is known regarding their ability to penetrate the COC. In the present study, the use of fluorescent particles allowed the detection of the presence of PS-MPs inside the ovine COC, specifically in CCs, where a significant increase in uptake was observed at the highest concentration. This result highlights that larger (microplastic) size particles are also able to penetrate the COC and not just PS-NPs, as already demonstrated in a previous study in the murine (36) and bovine (30 nm) (37) models. In agreement with our study, in the murine model, PS-NPs internalization did not extend beyond the CCs (36). Instead, in the bovine model, internalization occurred both in CCs and in the ooplasm, possibly via transcellular (CCs and transzonal projections) and paracellular (zona pellucida) routes (37). This could be due to a smaller particle size than those used in our study (30 nm rather than 200 nm) or to differences in zona pellucida composition/structure between ovine and bovine COCs. Furthermore, the evaluation of CCs grown in monolayers confirmed that PS-MPs bioaccumulation is time-dependent, starting after 6 h of culture and increasing after 24 h.

Given the accumulation of PS-MPs in CCs, further experiments were conducted to evaluate the impact on CC functionality. Previous *in vivo* studies reported increased expression of pro-apoptotic genes and decreased expression of those protecting from oxidative stress, thus resulting in apoptosis of granulosa cells, isolated from rats to which 500 nm PS-MPs were orally administered (38, 39). Our findings confirmed alterations in the expression of genes, related to oxidative stress and apoptosis, in CCs recovered from COCs exposed to PS-MPs during IVM. In detail, GPX, a gene essential in neutralizing peroxides and mitigating oxidative damage, and SOD, an enzyme converting superoxide radicals into hydrogen peroxide, showed downregulation at 100 μg/mL, thus indicating compromised antioxidant enzymatic response. The expression of CAT, an antioxidant gene responsible for converting hydrogen peroxide into water and oxygen, was not altered. Regarding the expression of genes involved in apoptosis, BAX, a pro-apoptotic gene, was upregulated at the lowest PS-MPs concentration, indicating an increased activation of apoptotic pathways. Conversely, the expression levels of BCL2, an anti-apoptotic gene, did not change across all treated groups.

In order to assess whether PS-MPs could affect CC viability, the TUNEL assay was used. This method is commonly employed to detect DNA fragmentation as one of the most specific markers in apoptosis, although also possibly linked to necrosis caused by toxic compounds or other insults (26). In our study, we highlighted significant increases in the rates of positive CCs at any tested concentration. Most of the studies conducted so far on the effects of MPs on germline cells report induction of the apoptosis process triggered by ROS overproduction (19). Thus, we hypothesized that the obtained results could be signs of an increase in the apoptotic index rather than necrosis. This observation aligns with a previous study in which increased apoptosis of granulosa cells was observed in rats treated with 500 nm PS-MPs compared to untreated animals (39). Overall, the combined results from real-time PCR for SOD, GPX, and BAX, and the TUNEL assay suggest that PS-MPs adversely impact CCs’ viability and functionality by inducing oxidative stress and apoptosis, in line with previous studies (19, 38).

Considering the intense bi-directional communication established between CCs and oocytes, within the COC structure, culminating in the acquisition of maturation and embryo developmental competence (40), this specific accumulation site could potentially lead to indirect oocyte damage resulting from CCs dysfunction and transzonal projections reduction, as already demonstrated (36, 41). In our experimental conditions, exposure to 50 and 100 μg/mL PS-MPs concentrations reduced the rate of mature oocytes. Concerning the higher tested concentration (100 μg/mL), our data are in agreement with those of previous *in vitro* studies in which reduced maturation rates were observed after exposure to 300 μg/mL of PS-MPs in porcine (42) as well as to 100 and 200 μg/mL of 100 nm PS-MPs in the bovine species (37). Conversely, another study in porcine oocytes showed a lack of toxicity after using 100 nm PS-MPs at 100 and 200 μg/mL (43). Regarding the intermediate concentration (50 μg/mL), the negative effect observed in our study is in line with data obtained in porcine oocytes exposed to 100 nm PS-MPs at a concentration of 30 and 50 μg/mL (42, 44). Instead, the exposure to our same particle concentration (50 μg/mL) and size did not exert any effect on bovine oocyte maturation (37). Finally, in our study, the lowest tested concentration (5 μg/mL) did not exert negative effects on oocyte maturation. In agreement with our data, no toxic effects were observed after exposure of porcine (42) and bovine (37) oocytes with 100 nm PS-MPs concentrated at 3 μg/mL and 5 μg/mL, respectively, in the two studies. Conversely, another study in the pig, also using 100 nm PS-MPs, highlighted a marked cytotoxic effect even at very low concentrations (1 and 10 μg/mL) (44). In addition, the negative impact observed after IVM is supported by an *in vivo* study, in which female mice treated with daily oral doses of 800 nm PS-MPs (30 mg/kg body weight for 35 days) (45) showed a significant decrease in the first PB extrusion rates compared to untreated animals, thus providing complementary evidence on the toxicological impact exerted by PS-MPs to *in vitro* studies.

In order to acquire embryo developmental competence, oocytes should reach not only nuclear but also cytoplasmic maturation, consisting of a remarkable reorganization of the ooplasm with important changes in structure, function, and/or distribution of all major organelles (46). Among them, mitochondria are the main site of ATP production, necessary for spindle assembly and chromosome segregation (47), and consequently of ROS, generated as by-products of oxidative phosphorylation mediated by the electron transport chain. When produced at low physiological levels, these molecules are important to trigger meiosis resumption and induce oocyte maturation (47, 48), but when they exceed oocyte antioxidant capacity, this could result in oxidative stress, potentially leading to macromolecular damage and impairment in oocyte function and viability. Therefore, mitochondrial quality determines the quality of the oocyte and the future embryo since they are exclusively maternally inherited (49). In our study, the rate of oocytes with perinuclear/subcortical mitochondrial distribution pattern, index of cytoplasmic maturation (24), did not vary at all PS-MP tested concentrations. This finding is not in agreement with a study in the porcine model (44) in which oocytes exposed to 100 nm PS-MPs displayed mitochondria dispersed throughout the cytoplasm, generally considered as indicative of cytoplasmic immaturity (46). These differences could be due to differences in methods used for mitochondria distribution staining and analysis. Instead, significantly increased ROS levels were observed in oocytes exposed to PS-MPs, in agreement with studies that tested PS-MPs ranging from 100 to 500 nm in bovine (37) and porcine (42–44) models, thus indicating the promotion of an oxidative stress condition.

Among cellular components that can be damaged by oxidative stress, the cytoskeleton appears to be particularly sensitive (31). The organization and dynamics of spindle microtubules and actin filaments are necessary for meiosis progression, polar body extrusion, and the distribution of some organelles, such as mitochondria (31). Our results revealed that PS-MP exposure disrupted chromosome alignment, meiotic spindle assembly, and cortical actin configuration, regardless of concentration, in agreement with a previous study in porcine oocytes (100 nm) (44). Instead, most of the control oocytes had chromosomes aligned on the meiotic spindle with a classical barrel-shape structure, with uniform intensity signals of actin filaments beneath the oolemma. Overall, our findings reveal that, regardless of PS-MP concentrations, those oocytes able to resume meiosis and reach the metaphase II stage are characterized by low quality due to oxidative stress and cytoskeletal disorganization, alterations that may impair their subsequent developmental competence.

Finally, to evaluate possible toxic carry-over effects on developmental competence derived from exposure to PS-MPs during IVM, oocytes were parthenogenetically activated, thus avoiding possible interfering sperm-related effects. At the concentration of 50 μg/mL, our findings confirmed the negative effects, already observed in relation to oocyte nuclear and cytoplasmic maturation, on embryo developmental competence, as embryo cleavage was slowed. Instead, at the lowest tested concentration (5 μg/mL), no toxic effects were evidenced on embryo cleavage as well as on blastocyst formation rate. To date, few controversial data have been published on the effects of MPs on embryo development. In the porcine model, a reduction in the blastocyst formation rate after exposure to 30 μg/mL PS-MPs during IVM and PA was observed (42). In another study in the same species, using 100 nm PS-MPs particles (43), no differences were evidenced in the blastocyst rates, not only at concentrations tested in the present study (5 and 50 μg/mL) but also at higher ones (100 and 200 μg/mL). In terms of blastocyst quality, the present study did not highlight any difference between treated and control groups, whereas in previous studies in the porcine species (42, 43), the blastocysts obtained displayed a reduced number of nuclei. Therefore, possible differences in the implantation rate would be interesting to investigate in the future in the ovine species. Moreover, with regard to *in vivo* studies, our data are in agreement with those of some authors reporting a lack of difference in the cleavage and blastocyst rate of PA oocytes among treated and untreated mice with 800 nm PS-MPs (45).

Overall, our results contribute to expanding our knowledge of the direct effects of PS-MPs on COC in a livestock animal model and confirm previous studies regarding their mechanisms of action based on the induction of oxidative stress and apoptosis. It is interesting to note that data on the effects of PS-MPs exposure during culture are controversial among different studies. This could be due to oocyte morpho-functional species-specific differences, as well as to the used culture conditions and methods. In some cases, differing results have been reported within the same species using identical PS-MP sizes and concentrations. Even in a controlled *in vitro* system, these effects may be partially attributable to endocrine-disrupting mechanisms, as PS-MPs have the potential to mimic natural hormones, antagonize their action, alter their metabolism, or modify the expression of specific receptors (50). Further studies are necessary to explore this hypothesis.

## 5 Conclusion

In conclusion, the present study provides new insights into the toxicity of PS-MPs on female reproduction, highlighting their negative effects on ovine COCs during IVM and providing insights into their action mechanisms. Our findings indicate that PS-MPs can penetrate and accumulate primarily in CCs, leading to their dysfunction, as evidenced by altered expression of genes related to oxidative stress and an increased rate of apoptosis. This, in turn, indirectly damages oocytes by reducing their quality and interfering with oocyte maturation and embryo development. Future research should focus on elucidating additional molecular pathways through which PS-MPs exert their toxic effects on fertility and reproduction and explore possible strategies to counteract them.

## Data Availability

The raw data supporting the conclusions of this article will be made available by the authors, without undue reservation.

## References

[1] Aardema H Dick Vethaak A Kamstra JH Legler J. Farm animals as a critical link between environmental and human health impacts of micro-and nanoplastics. Aardema et al. Microplastics Nanoplastics. (202) 4:5. doi: 10.1186/s43591-024-00082-w

[2] Mastrorocco A Temerario L Vurchio V Cotecchia S Martino NA Dell'Aquila ME. In vitro toxicity of a DEHP and cadmium mixture on sheep cumulus-oocyte complexes. Int J Mol Sci. (2024) 26:5. doi: 10.3390/ijms26010005, 39795862 PMC11719533

[3] Dell'Aquila ME Asif S Temerario L Mastrorocco A Marzano G Martino NA . Ochratoxin a affects oocyte maturation and subsequent embryo developmental dynamics in the juvenile sheep model. Mycotoxin Res. (2021) 37:23–37. doi: 10.1007/s12550-020-00410-y, 32996062 PMC7819917

[4] Wrzecińska M Kowalczyk A Cwynar P Czerniawska-Piątkowska E. Disorders of the reproductive health of cattle as a response to exposure to toxic metals. Biology. (2021) 10:882. doi: 10.3390/biology10090882, 34571759 PMC8467698

[5] Canipari R De Santis L Cecconi S. Female fertility and environmental pollution. Int J Environ Res Public Health. (2020) 17:8802. doi: 10.3390/ijerph17238802, 33256215 PMC7730072

[6] Guvvala PR Ravindra JP Selvaraju S. Impact of environmental contaminants on reproductive health of male domestic ruminants: a review. Environ Sci Pollut Res Int. (2020) 27:3819–36. doi: 10.1007/s11356-019-06980-4, 31845245

[7] Chiang C Mahalingam S Flaws JA. Environmental contaminants affecting fertility and somatic health. Semin Reprod Med. (2017) 35:241–9. doi: 10.1055/s-0037-1603569, 28658707 PMC6425478

[8] Martino NA Marzano G Mangiacotti M Miedico O Sardanelli AM Gnoni A . Exposure to cadmium during in vitro maturation at environmental nanomolar levels impairs oocyte fertilization through oxidative damage: a large animal model study. Reprod Toxicol. (2017) 69:132–45. doi: 10.1016/j.reprotox.2017.02.005, 28188904

[9] Pause FC Baufeld A Urli S Crociati M Stradaioli G Vanselow J . Exploring the influence of polystyrene-nanoplastics on two distinct in vitro systems in farm animals: a pilot study. Sci Total Environ. (2025) 976:179378. doi: 10.1016/j.scitotenv.2025.179378, 40209587

[10] Peng Y He Q. Reproductive toxicity and related mechanisms of micro(nano)plastics in terrestrial mammals: review of current evidence. Ecotoxicol Environ Saf. (2024) 279:116505. doi: 10.1016/j.ecoenv.2024.116505, 38810287

[11] Geng Y Liu Z Hu R Huang Y Li F Ma W . Toxicity of microplastics and nanoplastics: invisible killers of female fertility and offspring health. Front Physiol. (2023) 14:1254886. doi: 10.3389/fphys.2023.1254886, 37700763 PMC10493312

[12] Song X Du L Sima L Zou D Qiu X. Effects of micro(nano)plastics on the reproductive system: a review. Chemosphere. (2023) 336:139138. doi: 10.1016/j.chemosphere.2023.139138, 37285987

[13] Yang S Li M Kong RYC Li L Li R Chen J . Reproductive toxicity of micro- and nanoplastics. Environ Int. (2023) 177:108002. doi: 10.1016/j.envint.2023.108002, 37276763

[14] Yang J Kamstra J Legler J Aardema H. The impact of microplastics on female reproduction and early life. Anim Reprod. (2023) 20:e20230037. doi: 10.1590/1984-3143-AR2023-0037, 37547566 PMC10399130

[15] Chamas A Moon H Zheng J Qiu Y Tabassum T Jang JH . Degradation rates of plastics in the environment. ACS Sustain Chem Eng. (2020) 8:3494–511. doi: 10.1021/acssuschemeng.9b06635

[16] Hartmann NB Hüffer T Thompson RC Hassellöv M Verschoor A Daugaard AE . Are we speaking the same language? Recommendations for a definition and categorization framework for plastic debris. Environ Sci Technol. (2019) 53:1039–47. doi: 10.1021/acs.est.8b05297, 30608663

[17] Gigault J Halle AT Baudrimont M Pascal PY Gauffre F Phi TL . Current opinion: what is a nanoplastic? Environ Pollut. (2018) 235:1030–4. doi: 10.1016/j.envpol.2018.01.024, 29370948

[18] Afreen V Hashmi K Nasir R Saleem A Khan MI Akhtar MF. Adverse health effects and mechanisms of microplastics on female reproductive system: a descriptive review. Environ Sci Pollut Res Int. (2023) 30:76283–96. doi: 10.1007/s11356-023-27930-1, 37247153

[19] Ferrante MC Monnolo A Del Piano F Mattace Raso G Meli R. The pressing issue of Micro- and Nanoplastic contamination: profiling the reproductive alterations mediated by oxidative stress. Antioxidants. (2022) 11:193. doi: 10.3390/antiox11020193, 35204076 PMC8868557

[20] Adhikari M Biswas C Mazumdar P Sarkar S Pramanick K. Evaluating the potential of daily intake of polystyrene microplastics via drinking water in inducing PCOS and its ovarian fibrosis progression using female zebrafish. NanoImpact. (2024) 34:100507. doi: 10.1016/j.impact.2024.100507, 38663500

[21] Liu H Li H Liu Y Zhao H Peng R. Toxic effects of microplastic and nanoplastic on the reproduction of teleost fish in aquatic environments. Environ Sci Pollut Res Int. (2024) 31:62530–48. doi: 10.1007/s11356-024-35434-9, 39467868

[22] Sussarellu R Suquet M Thomas Y Lambert C Fabioux C Pernet ME . Oyster reproduction is affected by exposure to polystyrene microplastics. Proc Natl Acad Sci USA. (2016) 113:2430–5. doi: 10.1073/pnas.1519019113, 26831072 PMC4780615

[23] Temerario L Martino NA Bennink M de Wit A Hiemstra SJ Dell'Aquila ME . Effects of Cryoprotectant concentration and exposure time during Vitrification of immature pre-pubertal lamb cumulus-oocyte complexes on nuclear and cytoplasmic maturation. Animals. (2024) 14:2351. doi: 10.3390/ani14162351, 39199884 PMC11350855

[24] Temerario L Monaco D Mastrorocco A Martino NA Cseh S Lacalandra GM . New strategies for conservation of gentile di Puglia sheep breed, an autochthonous capital of millennial tradition in southern Italy. Animals. (2023) 13:2371. doi: 10.3390/ani13142371, 37508148 PMC10376504

[25] Martino NA Picardi E Ciani E D'Erchia AM Bogliolo L Ariu F . Cumulus cell transcriptome after cumulus-oocyte complex exposure to nanomolar cadmium in an in vitro animal model of prepubertal and adult age. Biology. (2023) 12:249. doi: 10.3390/biology12020249, 36829526 PMC9953098

[26] Majtnerová P Roušar T. An overview of apoptosis assays detecting DNA fragmentation. Mol Biol Rep. (2018) 45:1469–78. doi: 10.1007/s11033-018-4258-9, 30022463

[27] Mastrorocco A Martino NA Marzano G Lacalandra GM Ciani E Roelen BAJ . The mycotoxin beauvericin induces oocyte mitochondrial dysfunction and affects embryo development in the juvenile sheep. Mol Reprod Dev. (2019) 86:1430–43. doi: 10.1002/mrd.23256, 31410935

[28] Martino NA Lacalandra GM Filioli Uranio M Ambruosi B Caira M Silvestre F . Oocyte mitochondrial bioenergy potential and oxidative stress: within−/between-subject, in vivo versus in vitro maturation, and age-related variations in a sheep model. Fertil Steril. (2012) 97:720–8.e1. doi: 10.1016/j.fertnstert.2011.12.014 22260855

[29] Yang HW Hwang KJ Kwon HC Kim HS Choi KW Oh KS. Detection of reactive oxygen species (ROS) and apoptosis in human fragmented embryos. Hum Reprod. (1998) 13:998–1002. doi: 10.1093/humrep/13.4.998, 9619561

[30] Manders EMM Verbeek FJ Aten JA. Measurement of co-localization of objects in dual-colour confocal images. J Microsc. (1993) 169:375–82. doi: 10.1111/j.1365-2818.1993.tb03313.x, 33930978

[31] Piras AR Ariu F Maltana A Leoni GG Martino NA Mastrorocco A . Protective effect of resveratrol against cadmium-induced toxicity on ovine oocyte in vitro maturation and fertilization. J Anim Sci Biotechnol. (2022) 13:83. doi: 10.1186/s40104-022-00731-1, 35864507 PMC9306212

[32] Podda A Dujíčková L Ariu F Leoni GG Izquierdo D Paramio MT . Effect of liquid marble 3D culture system on in vitro maturation and embryo development of prepubertal goat oocytes. Animals. (2025) 15:188. doi: 10.3390/ani15020188, 39858188 PMC11758309

[33] Zhang R Wang M Chen X Yang C Wu L. Combined toxicity of microplastics and cadmium on the zebrafish embryos (*Danio rerio*). Sci Total Environ. (2020) 743:140638. doi: 10.1016/j.scitotenv.2020.140638, 32679492

[34] Leslie HA van Velzen MJM Brandsma SH Vethaak AD Garcia-Vallejo JJ Lamoree MH. Discovery and quantification of plastic particle pollution in human blood. Environ Int. (2022) 163:107199. doi: 10.1016/j.envint.2022.107199 35367073

[35] Zurub RE Cariaco Y Wade MG Bainbridge SA. Microplastics exposure: implications for human fertility, pregnancy and child health. Front Endocrinol. (2024) 14:1330396. doi: 10.3389/fendo.2023.1330396, 38239985 PMC10794604

[36] Xue Y Cheng X Ma ZQ Wang HP Zhou C Li J . Polystyrene nanoplastics induce apoptosis, autophagy, and steroidogenesis disruption in granulosa cells to reduce oocyte quality and fertility by inhibiting the PI3K/AKT pathway in female mice. J Nanobiotechnology. (2024) 22:460. doi: 10.1186/s12951-024-02735-7, 39090717 PMC11293132

[37] Merlo B Volsa AM Tovar L Gaiani M Gugole PM Attolini E . Effects of polystyrene nanoparticles on bovine oocyte in vitro maturation. Theriogenology. (2025) 244:117482. doi: 10.1016/j.theriogenology.2025.117482, 40381591

[38] An R Wang X Yang L Zhang J Wang N Xu F . Polystyrene microplastics cause granulosa cells apoptosis and fibrosis in ovary through oxidative stress in rats. Toxicology. (2021) 449:152665. doi: 10.1016/j.tox.2020.152665, 33359712

[39] Hou J Lei Z Cui L Hou Y Yang L An R . Polystyrene microplastics lead to pyroptosis and apoptosis of ovarian granulosa cells via NLRP3/Caspase-1 signaling pathway in rats. Ecotoxicol Environ Saf. (2021) 212:112012. doi: 10.1016/j.ecoenv.2021.112012, 33550074

[40] Turathum B Gao EM Chian RC. The function of cumulus cells in oocyte growth and maturation and in subsequent ovulation and fertilization. Cells. (2021) 10:2292. doi: 10.3390/cells10092292, 34571941 PMC8470117

[41] Zhang J Hu H Zhu Y Xin X Jin Y Zhao Q . Polystyrene/polylactic acid microplastics impair transzonal projections and oocyte maturation via gut microbiota-mediated lipoprotein lipase inhibition. J Hazard Mater. (2025) 496:139475. doi: 10.1016/j.jhazmat.2025.139475, 40784117

[42] Huang HM Peng HL Huang CM Zhang JT Li YH Lin ZL . Melatonin alleviates the damage of polystyrene microplastics to porcine oocytes by reducing oxidative stress and mitochondrial damage, and regulating autophagy and apoptosis levels. Animals. (2025) 15:3163. doi: 10.3390/ani15213163, 41227494 PMC12607468

[43] Spinaci M Dindo S Govoni N Tovar L Volsa AM Cappannari C . Effect of polystyrene nanoplastics on in vitro maturation of pig cumulus-encosed oocytes. Res Vet Sci. (2025) 197:105949. doi: 10.1016/j.rvsc.2025.105949, 41223719

[44] Cui Z Zhang J Zhang J Zhong J Wu J Miao Y . Microplastics (MPs) exposure impairs porcine oocyte quality by triggering oxidative stress-directed DNA damage and apoptosis with metabolomic alterations. Ecotoxicol Environ Saf. (2025) 300:118461. doi: 10.1016/j.ecoenv.2025.118461, 40472692

[45] Liu Z Zhuan Q Zhang L Meng L Fu X Hou Y. Polystyrene microplastics induced female reproductive toxicity in mice. J Hazard Mater. (2022) 424:127629. doi: 10.1016/j.jhazmat.2021.127629, 34740508

[46] Coticchio G Dal Canto M Mignini Renzini M Guglielmo MC Brambillasca F Turchi D . Oocyte maturation: gamete-somatic cells interactions, meiotic resumption, cytoskeletal dynamics and cytoplasmic reorganization. Hum Reprod Update. (2015) 21:427–54. doi: 10.1093/humupd/dmv011, 25744083

[47] Gualtieri R Kalthur G Barbato V Di Nardo M Adiga SK Talevi R. Mitochondrial dysfunction and oxidative stress caused by cryopreservation in reproductive cells. Antioxidants. (2021) 10:337. doi: 10.3390/antiox10030337, 33668300 PMC7996228

[48] Mateo-Otero Y Yeste M Damato A Giaretta E. Cryopreservation and oxidative stress in porcine oocytes. Res Vet Sci. (2021) 135:20–6. doi: 10.1016/j.rvsc.2020.12.024, 33418187

[49] van der Reest J Nardini Cecchino G Haigis MC Kordowitzki P. Mitochondria: their relevance during oocyte ageing. Ageing Res Rev. (2021) 70:101378. doi: 10.1016/j.arr.2021.101378, 34091076

[50] Campanale C Massarelli C Savino I Locaputo V Uricchio VF. A detailed review study on potential effects of microplastics and additives of concern on human health. Int J Environ Res Public Health. (2020) 17:1212. doi: 10.3390/ijerph17041212, 32069998 PMC7068600

